# WFG-RT-DETR: A Highly Robust Object Detection Model for Autonomous Vehicles in Adverse Weather Conditions

**DOI:** 10.3390/s26175475

**Published:** 2026-08-29

**Authors:** Xiaona Song, Jing Liu, Runqing Zhang, Lijun Wang

**Affiliations:** School of Mechanical Engineering, North China University of Water Resources and Electric Power, Zhengzhou 450045, China; songxiaona1@126.com (X.S.); rqing0208@163.com (R.Z.); wanglijun@ncwu.edu.cn (L.W.)

**Keywords:** adverse weather, object detection, RT-DETR, feature denoising, autonomous vehicles

## Abstract

Object detection under adverse weather conditions remains a challenging problem for autonomous driving systems due to image degradation caused by rain, snow, haze, and complex illumination. Although RT-DETR achieves a good balance between detection accuracy and efficiency, its performance is limited in adverse weather scenarios due to weather-induced feature distortion and unreliable query selection. In this paper, we propose WFG-RT-DETR, a robust real-time object detection framework for adverse weather conditions. A Weather-Focused Guidance (WFG) module is introduced to enhance feature representation by combining frequency-domain noise suppression with spatial structural compensation. Furthermore, a Weather-Aware Query Selection (WAQS) module is proposed to improve query initialization by incorporating weather-aware noise estimation, reducing false-positive proposals caused by environmental interference. An Exponential Moving Average (EMA) strategy is also employed to stabilize model training. Extensive experiments on the DAWN and WEDGE datasets demonstrate the effectiveness of the proposed method. Compared with RT-DETR, WFG-RT-DETR improves the mAP@50 on the DAWN dataset from 64.62% to 68.89% while maintaining real-time inference capability. Cross-dataset evaluations on the WEDGE dataset further verify its robustness and generalization under diverse adverse weather conditions. The proposed framework provides an effective solution for reliable object detection in intelligent transportation and autonomous driving applications.

## 1. Introduction

Object detection in the environmental perception of autonomous vehicles, enabling real-time identification of objects such as pedestrians, motor vehicles, and traffic signs, which directly determines the driving safety of autonomous vehicles. In recent years, with the rapid development of computer vision technology, the object detection capability of autonomous vehicles has been significantly improved. However, in real-world environments, adverse weather conditions such as fog, rain, snow, dust, and low-illumination environments can cause issues like enhanced background noise and reduced image contrast, interfering with object feature representation and consequently degrading the reliability of detection models in practical scenarios [1].

In recent years, deep learning-based object detection methods have achieved remarkable progress. Among them, the Transformer-based end-to-end detection framework RT-DETR [2] strikes a favorable balance between detection accuracy and inference speed by introducing a hybrid encoder and a query mechanism. Nevertheless, when directly applied to adverse weather scenes, it still exhibits certain limitations. First, environmental factors such as rain and snow textures and strong light reflections tend to generate abnormal responses during feature extraction, causing the model to learn noise information irrelevant to the objects. Second, the conventional query selection mechanism predominantly relies on classification confidence for candidate region screening, which is susceptible to interference from highlight regions and background textures in complex weather, leading to increased false detections. Moreover, adverse weather samples possess strong uncertainty, which also affects the stability of the model training process. To mitigate this, we adopt an exponential moving average strategy [3].

To address these issues, this paper proposes WFG-RT-DETR, a highly robust object detection model tailored for adverse weather scenes, using RT-DETR as the backbone. The proposed method improves the original network from three perspectives: feature enhancement, query optimization, and training stability. The main contributions are as follows:

1. We design a Weather-Focused Guidance (WFG) module. To tackle the aliasing between high-frequency noise and object edge information in adverse weather, a dual-branch structure combining frequency-domain noise suppression and spatial structure compensation is constructed. This design suppresses weather-induced noise while enhancing object contours and local textures, thereby improving feature representation capability in complex weather scenes.
2. We propose a Weather-Aware Query Selection (WAQS) mechanism. To address the problem that the query selection process in RT-DETR is easily disturbed by rain and snow reflections and false background responses, a weather noise estimation branch is introduced to dynamically calibrate the classification scores of candidate queries, thus improving the quality of initial queries and reducing the false detection rate.
3. We introduce an Exponential Moving Average (EMA) training strategy [3]. By maintaining a moving average of model parameters, the training fluctuations caused by complex weather samples are mitigated, improving model convergence stability and cross-domain generalization capability.
4. We conduct extensive experiments on the DAWN adverse weather dataset [4] and the cross-domain WEDGE test set [5]. Combined with feature response visualization analysis, the results demonstrate that the proposed method can effectively suppress environmental noise interference and enhance detection performance under adverse weather conditions.

The remainder of this paper is organized as follows: Section 2 reviews the current research status of object detection and object detection in adverse weather. Section 3 elaborates on the overall framework of WFG-RT-DETR and the design principles of each module. Section 4 verifies the effectiveness of the proposed method through comparative experiments, ablation studies, and feature visualization experiments. Section 5 concludes the paper and discusses future research directions.

## 2. Related Work

### 2.1. Object Detection Methods

As a fundamental task in computer vision, object detection has evolved from traditional handcrafted features to deep learning-based approaches. Early methods primarily relied on manually designed features, such as HOG and SIFT, which exhibited limited robustness in complex environments. With the advancement of deep learning, convolutional neural network (CNN)-based detection methods have gradually become mainstream.

Girshick et al. proposed Faster R-CNN [6], which achieves object localization and classification through a Region Proposal Network (RPN), improving detection accuracy but with a relatively complex detection pipeline. Subsequently, Redmon et al. introduced the YOLO series [7], which reformulates the detection task as an end-to-end regression problem and has been widely adopted in real-time detection applications. Subsequent YOLO variants, including YOLOv3 [8] and YOLOv7 [9], further improved detection performance (see also the comprehensive review [10]).

In recent years, Transformers have been introduced into the object detection field. Carion et al. proposed DETR [11], enabling Transformer-based end-to-end detection while avoiding the need for hand-designed anchor boxes. However, it suffers from slow training convergence and inadequate detection of small objects. Zhu et al. proposed Deformable DETR [12], which enhances multi-scale feature modeling capability through deformable attention. Conditional DETR [13] further accelerates training convergence. Building upon these works, RT-DETR [2] further optimizes the Transformer detection architecture by introducing a Hybrid Encoder for efficient feature fusion, achieving a favorable balance between detection accuracy and real-time performance.

Nevertheless, RT-DETR is primarily designed for generic scenes and is susceptible to factors such as rain and snow noise and illumination variations in adverse weather environments. Therefore, how to enhance the model’s adaptability to complex weather features becomes the focus of this study.

### 2.2. Object Detection Methods in Adverse Weather

To address the problem of image degradation caused by adverse weather, recent research has primarily focused on two directions: image enhancement and detection network optimization, aiming to improve the robustness of object detection models in complex weather environments.

The first category of methods improves the quality of input images through image enhancement. For example, the Dark Channel Prior (DCP) dehazing algorithm proposed by He et al. [14] can effectively restore the contrast and clarity of foggy images. Subsequently, numerous deep learning-based dehazing methods [15,16], deraining methods [17,18], and desnowing methods [19,20,21] have been proposed. However, such methods generally require an additional independent image restoration network, incurring significant computational overhead. Moreover, the restoration process may introduce pseudo-textures or cause the loss of certain object details, adversely affecting subsequent detection results.

The other category of methods directly enhances feature representation capability from within the detection network. For instance, the Squeeze-and-Excitation Network (SENet) proposed by Hu et al. [22] adaptively enhances important features and suppresses redundant information through a channel attention mechanism. The Convolutional Block Attention Module (CBAM) proposed by Woo et al. [23] further integrates channel attention and spatial attention based on SENet, thereby effectively improving object localization accuracy and detection robustness.

In addition, frequency-domain analysis methods have been increasingly applied to adverse weather vision tasks in recent years. Qin et al. proposed FFA-Net [24], which restores image details through feature fusion and attention mechanisms. Yang et al. proposed Fourier Domain Adaptation (FDA) [25], which utilizes Fourier transform for frequency-domain style transfer. Chi et al. introduced Fast Fourier Convolution (FFC) [26], which incorporates Fourier transform into convolutional networks. Rao et al. proposed Global Filter Networks (GFNet) [27], which leverage frequency-domain global filtering to enhance feature representation. Recently, FDA-DETR [28] and Frequency-Domain Cross-Attention (FCAT) [29] have further explored frequency-domain mechanisms for robust detection in adverse weather. Frequency-guided sparse fusion algorithms [30] have also been applied to multispectral object detection in complex environments.

For low-illumination conditions, Zero-Reference Deep Curve Estimation (Zero-DCE) [31] and LIME [32] have been proposed to enhance low-light images without requiring paired training data. For domain adaptation in adverse weather, Chen et al. [33] proposed a domain adaptive Faster R-CNN framework to bridge the domain gap between clear and adverse weather images. Several large-scale driving datasets, including BDD100K [34] and Foggy Cityscapes [35], have been established. The KITTI dataset [36] is also widely used in this area.

Furthermore, several recent works have developed dedicated object detectors specifically designed for adverse weather conditions. For instance, AWD-YOLO [37] incorporates weather-adaptive mechanisms into the YOLO framework, while Image-Adaptive YOLO [38] adapts image features on the fly for robust detection. Kumar et al. [39] explored data merging strategies combined with YOLOv8 for adverse weather detection. CW-DETR [40] focuses on traffic sign detection in complex weather, and FAD-DETR [41] introduces frequency-adaptive and haze-aware mechanisms for autonomous driving. PGEM-DETR [42] employs physics-guided enhancement for drone-based detection. Weather-robust insulator detectors [43] target specific infrastructure applications, and ECL-YOLOv11 [44] enhances automotive vision systems under adverse weather. Özcan et al. [45] utilized metaheuristic algorithms to improve YOLO detection performance in adverse weather.

Peng et al. [46] recently proposed MTW-DETR, a multi-task collaborative optimization framework for adverse-weather object detection. The method employs a dual-stream architecture to jointly optimize image restoration and object detection, allowing restoration-related information to be shared with the detection branch. It further introduces feature enhancement modules to improve representation under degraded weather conditions. Experiments on the real-world RTTS fog dataset and synthetic adverse-weather datasets demonstrate the effectiveness of the joint restoration-detection strategy.

These studies indicate that leveraging attention mechanisms and frequency-domain information can effectively enhance feature representation capability. In this paper, frequency-domain analysis methods are introduced into the detection network to suppress weather-induced noise at the feature level, while spatial structure compensation is incorporated to strengthen object features.

### 2.3. Query Selection and Model Optimization Methods

In Transformer-based detection frameworks, the quality of queries directly determines detection performance. DETR [11] accomplishes detection through object queries, while RT-DETR [2] further adopts a Top-K strategy to select high-response regions as initial queries, thereby improving detection efficiency. However, under adverse weather conditions, rain and snow reflections and complex backgrounds can generate abnormally high-response regions, allowing erroneous queries to enter the decoder stage.

To address similar issues, Li et al. proposed DN-DETR [47], which introduces denoising queries to accelerate training convergence and improve query stability. Liu et al. proposed DINO [48], which further combines denoising training with anchor box initialization, effectively enhancing object localization accuracy and detection performance in complex scenes. Moreover, several studies on dynamic query optimization, including layer-wise query selection [49] and adaptive query selection (AQS-IDETR) [50], reduce the number of invalid queries entering the decoder. Other strategies, such as rethinking query selection [49], Compact DETR [51], and CCDN-DETR [52] for SAR detection, further improve detection robustness.

Furthermore, to address training fluctuations caused by complex weather data, this paper introduces an Exponential Moving Average (EMA) strategy. The classical EMA formulation [3] and its application in semi-supervised learning [53] have demonstrated its effectiveness in stabilizing training and improving generalization.

In summary, existing methods have achieved certain progress in object detection, feature enhancement, and model optimization. However, challenges such as pronounced weather noise interference, query selection susceptibility to background influence, and insufficient training stability remain. Therefore, this paper proposes the WFG-RT-DETR model by integrating frequency-domain feature analysis, attention enhancement, query optimization, and the EMA training strategy. The proposed method improves feature robustness against interference through the WFG module, optimizes detection queries via WAQS, and enhances training stability using EMA, thereby strengthening detection performance in adverse weather scenes.

## 3. Method

### 3.1. Overall Architecture

**Figure 1:**
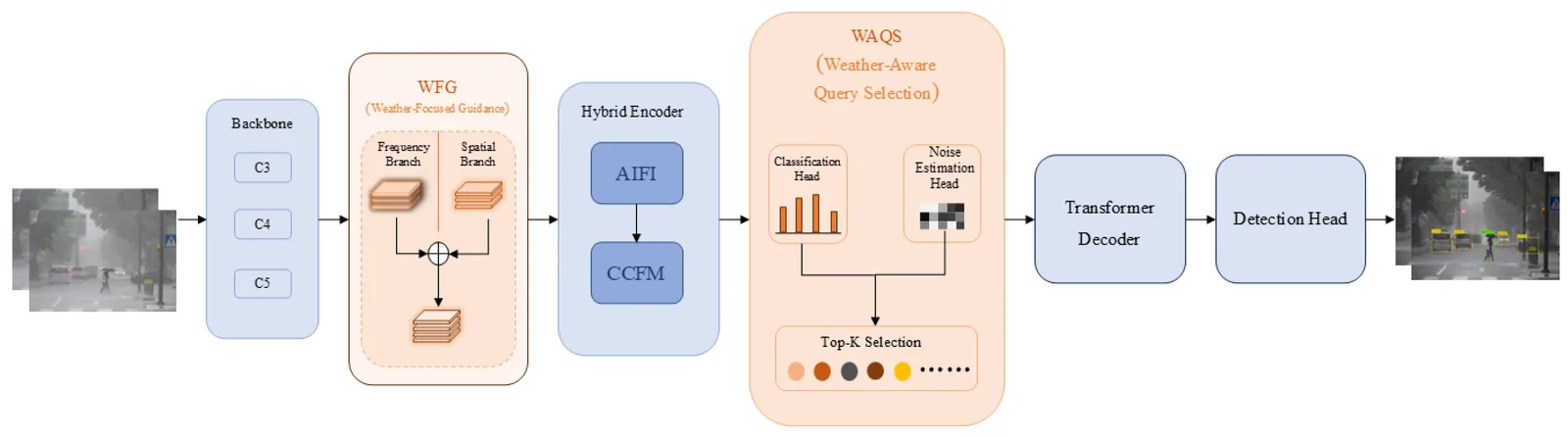
Overall architecture of the WFG-RT-DETR model. Built upon RT-DETR, the model embeds a Weather-Focused Guidance (WFG) module within the Hybrid Encoder, introduces a Weather-Aware Query Selection (WAQS) mechanism at the decoder input, and adopts an Exponential Moving Average (EMA) strategy to stabilize training. The figure clearly illustrates the complete forward path from backbone feature extraction to the detection head output.

The overall architecture of the proposed WFG-RT-DETR is illustrated in Figure 1. Built upon the RT-DETR [2] framework, the model optimizes the feature extraction and detection prediction processes to address issues such as image quality degradation, background noise interference, and weakened object features under adverse weather conditions. The entire network maintains an end-to-end detection pipeline, which primarily consists of four stages: input image feature extraction, weather-aware feature enhancement, candidate query optimization, and object prediction.

First, the input images from adverse weather scenes undergo multi-scale feature extraction through the backbone network, yielding feature representations containing information at different semantic levels. Due to factors such as rain, snow, haze, and low illumination, shallow features often contain more local noise responses, while deep features may suffer from blurred object edges and weakened semantic information. To address this, a Weather-Focused Guidance (WFG) module is introduced into the feature fusion stage of the RT-DETR hybrid encoder to further refine the extracted multi-scale features. This module suppresses abnormal responses caused by weather degradation through frequency-domain information modeling, while simultaneously compensating for object contour information via a spatial structure enhancement branch, thereby improving the effectiveness of feature representations.

Second, the WFG-refined features are fed into the hybrid encoder for cross-scale fusion and subsequently enter the decoding stage. To tackle the problem that environmental factors such as strong light reflections and rain and snow textures tend to generate erroneous candidate regions under adverse weather, a Weather-Aware Query Selection (WAQS) module is designed. This module leverages the enhanced feature information to re-evaluate the initial object queries, reducing misleading responses induced by environmental noise through adjusting the confidence scores of candidate locations, thus enabling the decoder to focus more on genuine object regions.

Finally, the optimized query features are passed into the detection head for class prediction and bounding box regression. To mitigate gradient fluctuations caused by complex weather samples during training, an Exponential Moving Average (EMA) strategy [3] is further employed to smooth the model parameter update process, enhancing the stability of network training.

In summary, WFG-RT-DETR improves upon the original RT-DETR [2] from four aspects: feature extraction, feature enhancement, query selection, and parameter optimization. This enables the model to improve object detection robustness in complex weather environments while preserving the advantages of end-to-end real-time detection.

### 3.2. Weather-Focused Guidance (WFG) Module

**Figure 2:**
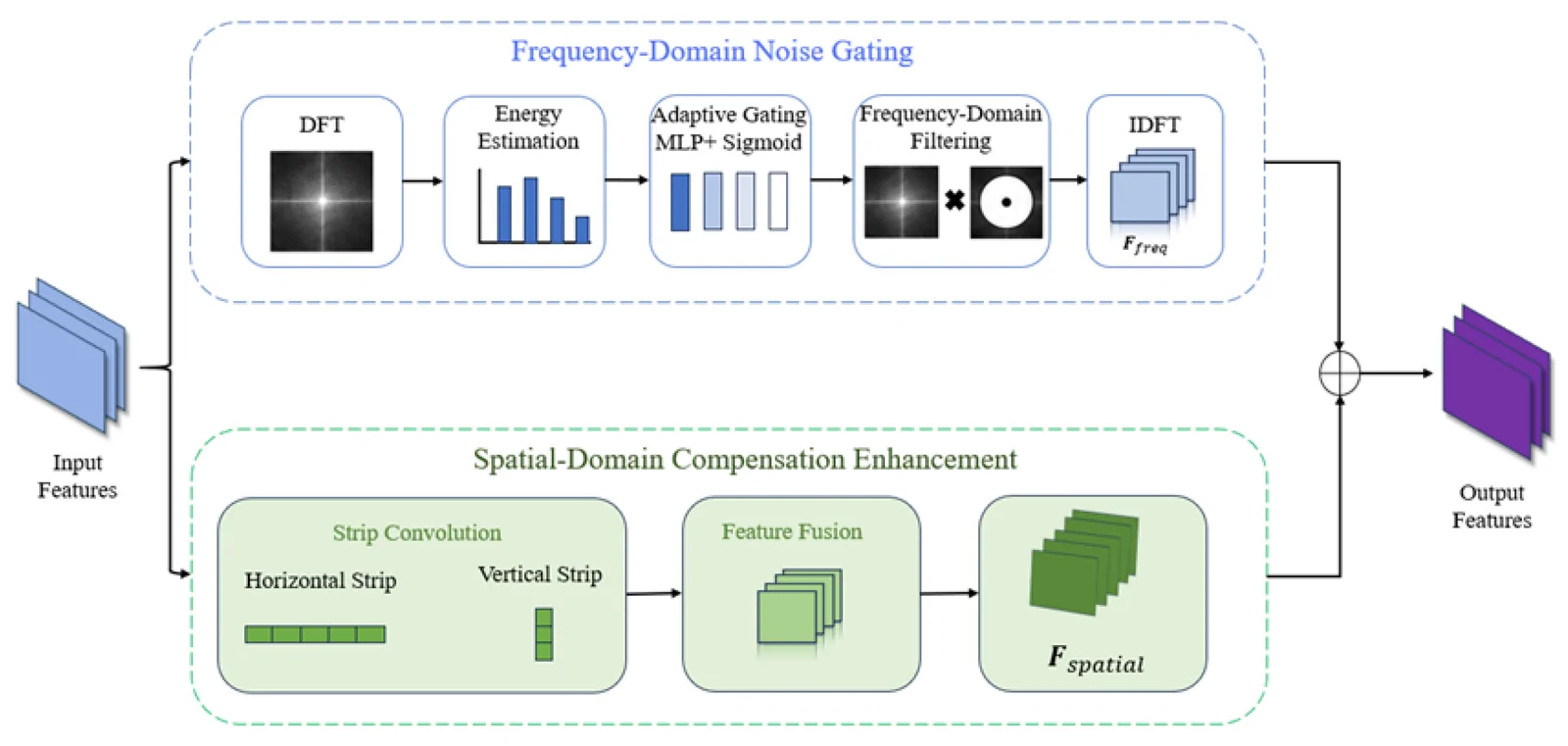
Structure of the Weather-Focused Guidance (WFG) module. The WFG module adopts a dual-branch design that combines frequency-domain noise suppression with spatial-domain compensation. The frequency-domain branch filters out high-frequency weather noise through DFT, energy estimation, an adaptive gating MLP, and IDFT. The spatial branch reconstructs local contours using horizontal strip convolution (1×7) and vertical strip convolution (7×1). The outputs of the two branches are summed to produce the purified features.

Under adverse weather conditions, image feature degradation exhibits pronounced discrepancies in the frequency-domain response. In real-world road scenes, discrete weather disturbances such as rain streaks, snow particles, and dust cause abrupt local brightness variations, which manifest as strong anomalous high-frequency responses when mapped into the feature space. Conversely, dense fog and low-illumination environments lead to an overall decline in image contrast and blurred object edge structures, essentially weakening the effective frequency components that characterize the fine structures of objects. In terms of the frequency-domain feature distribution, conventional traffic objects exhibit broadband spectral characteristics: low-frequency components correspond to the global contour and macroscopic structure of objects, while high-frequency components carry edge details and texture information. However, weather-induced degradation factors such as rain and snow textures and local specular reflections primarily exist in the form of anomalous high-frequency signals. Traditional convolution operations based on image pyramids perform indiscriminate aggregation of features across the entire frequency band, making it difficult to distinguish between the high-frequency details intrinsic to objects and weather-induced noise signals. This easily leads to irreversible aliasing of the two, consequently degrading the feature representation capability. To address this problem of irreversible aliasing between high-frequency noise and object features, this paper proposes the Weather-Focused Guidance (WFG) module, which adopts a dual-branch parallel processing architecture combining frequency-domain adaptive noise suppression with spatial structure compensation. The detailed structure is illustrated in Figure 2. The input features are fed into two parallel branches: the frequency-domain branch adaptively modulates channel responses according to their frequency-domain energy, thereby reducing the influence of anomalous responses associated with weather degradation, while the spatial branch enhances the feature representation of object contours and local structures using strip convolution. Finally, the outputs of the two branches are fused via element-wise summation to produce a high-quality feature representation with noise suppression and structural restoration.

#### 3.2.1. Frequency-Domain Noise Gating

Under adverse weather conditions, environmental factors such as rain, snow, haze, and light reflections can significantly interfere with object detection. Specifically, weather noise like raindrops and snowflakes typically manifests as locally abrupt textural information, which tends to generate anomalous high-frequency responses during feature extraction. However, object regions themselves also contain abundant high-frequency detail information, such as vehicle edges, tire structures, and object textures. Therefore, simply suppressing all high-frequency information would lead to the loss of effective object details.

Traditional spatial-domain convolution-based feature extraction methods primarily aggregate information within local regions, making it difficult to distinguish between high-frequency details originating from objects and noise responses caused by weather factors when facing complex weather scenes. This easily leads to the intermixing of the two types of information, reducing the effectiveness of feature representations. To address this, this paper introduces a frequency-domain analysis mechanism that maps features from the spatial domain to the frequency domain, enabling the network to analyze different response components from the perspective of frequency distribution and adaptively regulate anomalous activations potentially induced by weather degradation based on their frequency response characteristics.

For the multi-scale feature map $X\in R^{C\times H\times W}$ output by the backbone, a two-dimensional Discrete Fourier Transform (DFT) is first applied to each channel along the spatial dimensions, converting the spatial-domain features into frequency-domain representations:

(1) $$F\left(u,v\right)=F\left(X\right)=\sum_{h=0}^{H-1}\sum_{w=0}^{W-1}X\left(h,w\right)e^{-j2\pi (\frac{uh}{H}+\frac{vw}{W})}$$

where, F(·) denotes the two-dimensional Fourier transform operation, *H* and *W* represent the height and width of the feature map at the current scale, respectively, and X(h,w) denotes the feature value of the input feature map at coordinates (h,w).

To enable adaptive channel-wise adjustment, this paper computes the frequency-domain energy of each channel as a descriptor of its frequency response and uses this information to generate the corresponding gating weights. Specifically, the spatial features are first mapped to the frequency domain via DFT, where $F_{c}\left(u,v\right)$ denotes the response of the *c*-th channel at frequency position (u,v). The channel-wise frequency-domain energy $E_{c}$ is obtained by accumulating the squared magnitudes over all frequency positions and performing spatial normalization. This energy characterizes the overall spectral response strength of each channel and provides a basis for adaptive channel-wise frequency-domain modulation. By assigning different weights to different channels, the proposed mechanism reduces the contribution of channels with potentially strong weather-induced responses while retaining channels that contain useful object-related information. The calculation formula is as follows:

(2) $$E_{c}=\frac{1}{H\times W}\sum_{u=0}^{H-1}\sum_{v=0}^{W-1}{\left|F_{c}\left(u,v\right)\right|}^{2}$$

where $E_{c}$ denotes the frequency-domain energy of the *c*-th channel, $F_{c}\left(u,v\right)$ represents the spectral feature of the *c*-th channel at frequency coordinate (u,v), and *H* and *W* denote the height and width of the feature map, respectively.

Subsequently, a multi-layer perceptron (MLP) composed of two 1×1 convolutions maps the channel-wise frequency-domain energy feature *E* into an adaptive gating mask $M\in {\left[0,1\right]}^{C\times 1\times 1}$:

(3) $$M=\sigma \left({\mathrm{Conv}}_{2}\left(\mathrm{ReLU}\left({\mathrm{Conv}}_{1}\left(E\right)\right)\right)\right)$$

The gating mask adaptively assigns a weight to each channel according to its spectral energy, enabling differentiated modulation of the frequency-domain responses across channels. The generated channel-wise gating mask is then applied to the original frequency-domain features. Since the gating weight is shared across the frequency coordinates within each channel, this operation performs adaptive channel-level modulation of the entire spectral response. In this way, channels with stronger anomalous responses induced by weather degradation can be down-weighted, while useful frequency information associated with object structures is retained.

(4) $${\hat{F}}_{c}\left(u,v\right)=F_{c}\left(u,v\right)⊙M_{c}$$

where ⊙ denotes element-wise multiplication, $F_{c}\left(u,v\right)$ represents the frequency-domain response of the *c*-th channel, and $M_{c}$ denotes the corresponding channel-wise gating weight. The frequency-modulated features are then transformed back to the original spatial domain via a two-dimensional inverse discrete Fourier transform (IDFT), yielding the output features of the frequency-domain branch, denoted as $F_{freq}$:

(5) $$F_{freq}=F^{-1}\left({\hat{F}}_{c}\left(u,v\right)\right)$$

where, $F^{-1}\left(\cdot \right)$ denotes the two-dimensional inverse discrete Fourier transform operator. Compared with conventional hard-threshold low-pass filtering with fixed cutoffs, the smooth parameter update mechanism of this adaptive gating avoids irreversible loss of valid information. Compared with fixed hard-threshold filtering, the proposed adaptive channel-wise modulation does not impose a manually predefined frequency cutoff. Instead, it adjusts the contribution of each channel according to its spectral response, which helps reduce weather-induced interference while avoiding excessive suppression of useful feature information, including object contours and local textures.

#### 3.2.2. Spatial Receptive Field Compensation and Enhancement

While frequency-domain noise suppression effectively filters out high-frequency weather interference, it may also cause excessive feature smoothing, resulting in the loss of object edge contours and local structural information. To address this, a spatial-domain compensation and enhancement branch is constructed, which reconstructs object structural information through two cascaded modules: strip convolution feature extraction and feature fusion.

1. Strip Convolution Feature Extraction: Asymmetric strip convolutions are adopted in place of conventional square convolutions. Specifically, a horizontal 1×7 convolution and a vertical 7×1 convolution are set up in parallel to extract features from two orthogonal directions. Compared with square convolutions of equivalent receptive field and strip convolutions better adapt to the shapes of objects with significant aspect ratios in traffic scenes, such as pedestrians and pole-like facilities, enabling precise capture of edge textures. Moreover, they can extend long-range contextual associations and repair broken contours while reducing the introduction of irrelevant background noise and lowering computational redundancy.
2. Feature Fusion: The output features from the horizontal and vertical strip convolutions are fused to integrate the structural information from both orthogonal directions, yielding the final output features of the spatial branch, denoted as $F_{spatial}$. This process aggregates multi-dimensional edge and contour information, which strengthens the continuous structural representation of objects, compensates for the detail smoothing loss caused by frequency-domain denoising, and improves the feature discriminability of low-contrast objects and small objects.

Finally, the denoised features from the frequency-domain branch and the structurally enhanced features from the spatial branch are summed element-wise to produce the final output features of the WFG module, denoted as $F_{out}$:

(6) $$F_{out}=F_{freq}+F_{spatial}$$

### 3.3. Weather-Aware Query Selection (WAQS)

Although the WFG module can effectively suppress high-frequency noise caused by adverse weather such as rain, snow, and haze between the backbone and the hybrid encoder, and enhance the feature representation capability of object regions, object detection performance depends not only on feature quality but also on the quality of the initial object queries in the Transformer decoder. RT-DETR [2] adopts a Top-K Query Selection strategy, which selects the *K* candidate positions with the highest classification scores based on the encoder output features as decoder input. This strategy is computationally simple and fast in inference, yet it relies entirely on classification responses and lacks the ability to discriminate weather-induced noise. Under adverse weather conditions, factors such as rain and snow textures, water reflections, and haze occlusion can easily cause background regions to produce abnormally high responses, while the classification scores of genuine objects decrease due to occlusion or blurring. Consequently, erroneous queries enter the decoder, increasing the false detection rate and degrading detection accuracy. Therefore, query selection based solely on classification scores is insufficient to meet the requirements of object detection in complex weather scenes.

**Figure 3:**
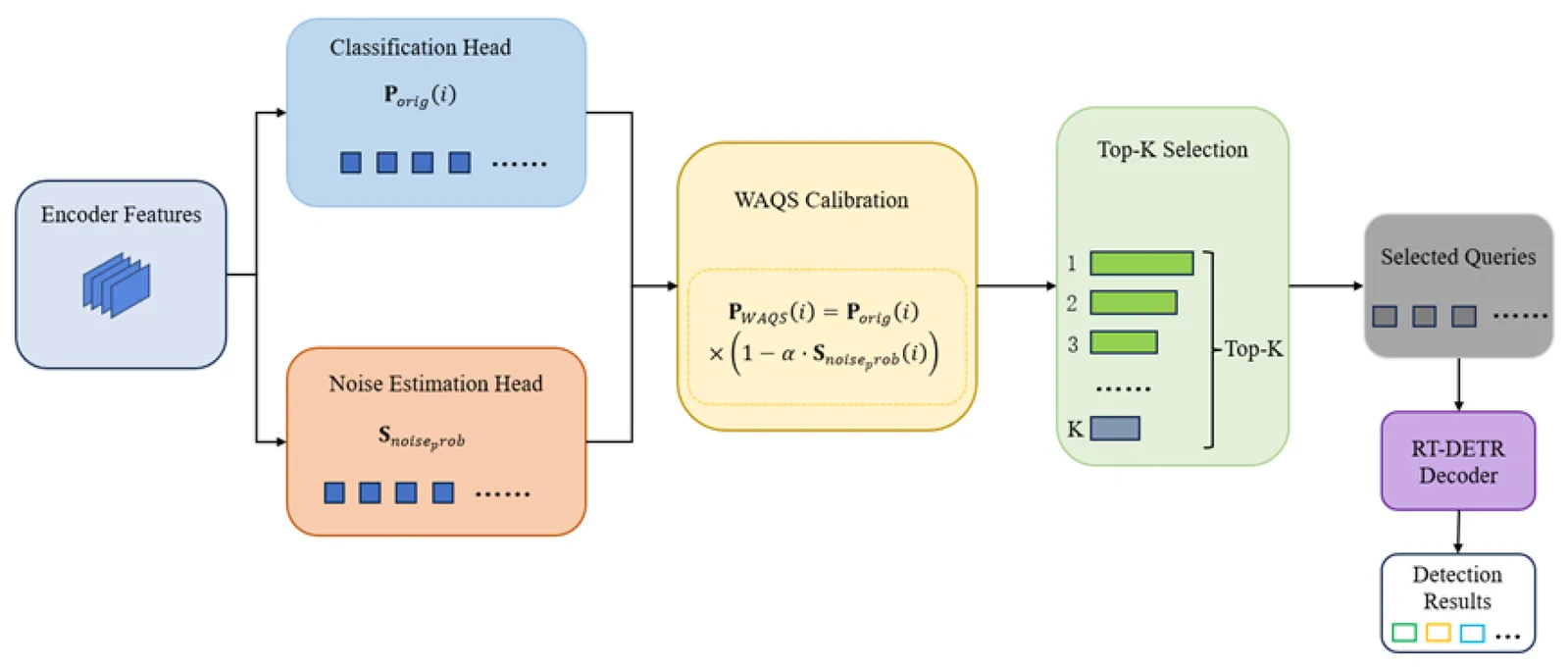
Schematic diagram of the Weather-Aware Query Selection (WAQS) mechanism.

To address this problem, a Weather-Aware Query Selection (WAQS) mechanism is introduced to incorporate weather-interference information into the query selection process. As shown in Figure 3, the encoder features are processed by a Classification Head and a Noise Estimation Head. The Classification Head produces the original classification score $P_{orig}\left(i\right)$, while the Noise Estimation Head generates a weather-interference response $S_{noise\_prob}\left(i\right)$ for each candidate position *i*. A higher value indicates stronger weather-related interference, such as rain and snow textures, haze, or water reflections. The weather-interference response is used as an auxiliary signal for query calibration rather than as an independently annotated weather-noise classification task. Therefore, the proposed mechanism does not require additional manually annotated weather-noise regions.

The Noise Estimation Head shares the same backbone and encoder features with the Classification Head and is jointly optimized within the overall end-to-end detection framework. No independent noise-estimation loss or additional weather-noise annotations are required. Its parameters are updated through the overall detection objective, allowing the head to implicitly learn feature responses associated with weather interference from the training data.

Based on the estimated weather-interference response, WAQS recalibrates the original classification score as follows:

(7) $$P_{WAQS}\left(i\right)=P_{orig}\left(i\right)\times \left(1-\alpha \cdot S_{noise\_prob}\left(i\right)\right)$$

where $P_{WAQS}\left(i\right)$ denotes the weather-aware calibrated classification score, $P_{orig}\left(i\right)$ denotes the original classification score, $S_{noise\_prob}\left(i\right)$ denotes the estimated weather-interference response at candidate position *i*, and α denotes the weather-interference penalty coefficient that controls the suppression strength.

In this study, the penalty coefficient α is empirically set to 0.5 and kept fixed throughout the experiments. This value provides a moderate suppression strength, reducing weather-related responses while avoiding excessive suppression of potentially valid object responses.

After score calibration, Top-K Query Selection is performed according to $P_{WAQS}$, and the *K* highest-scoring candidates are selected as the initial object queries for the RT-DETR decoder. Since the calibrated scores incorporate both object-related and weather-interference information, weather-affected background responses are suppressed during query selection, reducing their influence on subsequent decoding. Overall, WAQS introduces a lightweight weather-aware calibration process before Top-K selection without changing the original end-to-end detection pipeline, thereby improving the quality of initial object queries and the robustness of detection under adverse weather conditions.

### 3.4. EMA Parameter Smoothing Mechanism

Since adverse weather object detection data typically exhibit substantial scene variations—such as varying degrees of rain and snow, object visibility, and ambient brightness—these factors lead to a more complex distribution of training samples, making the model weight update process vulnerable to anomalous samples and causing a certain degree of weight oscillation.

To address this issue, this paper incorporates an Exponential Moving Average (EMA) strategy [3] into WFG-RT-DETR to improve the stability of the model training process. It should be noted that EMA does not participate in the forward inference process of the network, nor does it alter the model architecture. Rather, it maintains an additional copy of the model parameters during the training phase, obtaining smoother parameters through a weighted average of historical and current parameters. The EMA strategy has also been successfully applied in semi-supervised medical image segmentation [53], demonstrating its effectiveness in stabilizing training.

The EMA strategy performs a dynamically smoothed update of model parameters during training. By fusing the current iteration parameters with their historical moving average, it mitigates the drastic fluctuations caused by individual gradient updates, leading to more stable weight changes. Its parameter update process can be expressed as:

(8) $$\theta_{ema}=\beta \theta_{ema}+\left(1-\beta \right)\theta_{current}$$

where $\theta_{current}$ denotes the model parameters after the gradient update at the current training step, $\theta_{ema}$ represents the smoothed parameters maintained by EMA, and β is the smoothing coefficient that controls the influence of historical parameters.

During training, EMA continuously maintains an exponential moving average of the model parameters. After training, the smoothed parameters maintained by EMA are adopted as the final model weights for inference, thereby reducing the impact of parameter oscillations during training on model performance.

Through the exponential moving average mechanism, the model mitigates drastic weight fluctuations caused by individual gradient updates from abnormal samples, leading to a more stable training process.

## 4. Experiments

### 4.1. Experimental Setup and Evaluation Metrics

To validate the effectiveness of the proposed WFG-RT-DETR model for object detection under adverse weather conditions, systematic experiments are conducted on the DAWN adverse weather object detection dataset [4] in this chapter. Furthermore, to verify the generalization capability of the model across different weather environments, transfer experiments are performed on the WEDGE cross-domain test set [5].

All experiments are conducted on the DAWN dataset, which contains 1027 images in total, with a 70/20/10 random split for training, validation, and testing, resulting in 718, 205, and 104 images, respectively. Input images are resized to 640×640 pixels. Both the RT-DETR baseline and WFG-RT-DETR are trained using the AdamW optimizer with $\beta_{1}=0.9$, $\beta_{2}=0.999$, an initial learning rate of $5\times 10^{-5}$, weight decay of $1\times 10^{-4}$, and a batch size of 4 for training and 8 for validation and testing. The learning rate is decayed by a factor of 0.1 at epochs 48 and 64 using the MultiStepLR scheduler, and the total number of training epochs is set to 72. Gradient clipping with a maximum norm of 0.5 is applied to stabilize training. For WFG-RT-DETR, the EMA decay coefficient is set to 0.9999 with 2000 warmup steps. Data augmentation for training includes random photometric distortion (*p* = 0.8), random horizontal flipping (*p* = 0.5), and resizing to 640 pixels. No augmentation is applied during validation or testing. All models are trained using AMP mixed precision on a single NVIDIA GeForce RTX 4090 GPU with 24GB memory. Inference is performed in FP32 precision. All splits are generated with a fixed random seed to ensure reproducibility.

#### 4.1.1. Dataset

**Figure 4:**
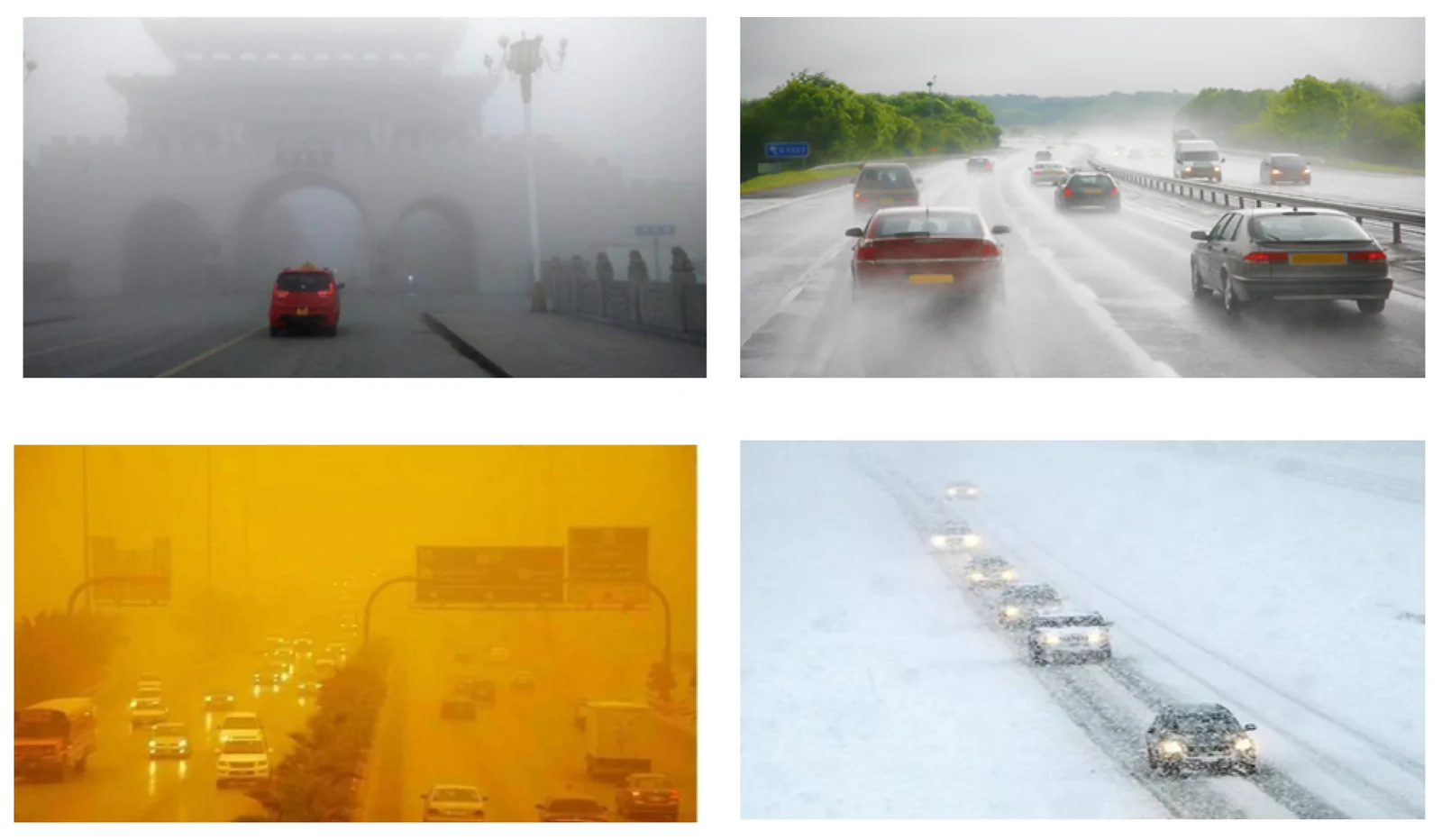
Example images from the DAWN dataset under four adverse weather conditions: fog, rain, sand, and snow.

The experiments in this paper primarily employ the DAWN (Detection in Adverse Weather Nature) dataset [4] for model training and testing. DAWN is a real-world adverse weather object detection dataset containing a total of 1027 real traffic scene images, covering four typical adverse weather conditions: fog, rain, snow, and sandstorm, as shown in Figure 4. The dataset annotates six categories of traffic objects: person, bicycle, car, motorcycle, bus, and truck. Since the images commonly suffer from issues such as blurred object edges, reduced contrast, loss of texture details, and increased background interference, the dataset can effectively evaluate the detection performance and robustness of object detection models in complex weather environments.

**Figure 5:**
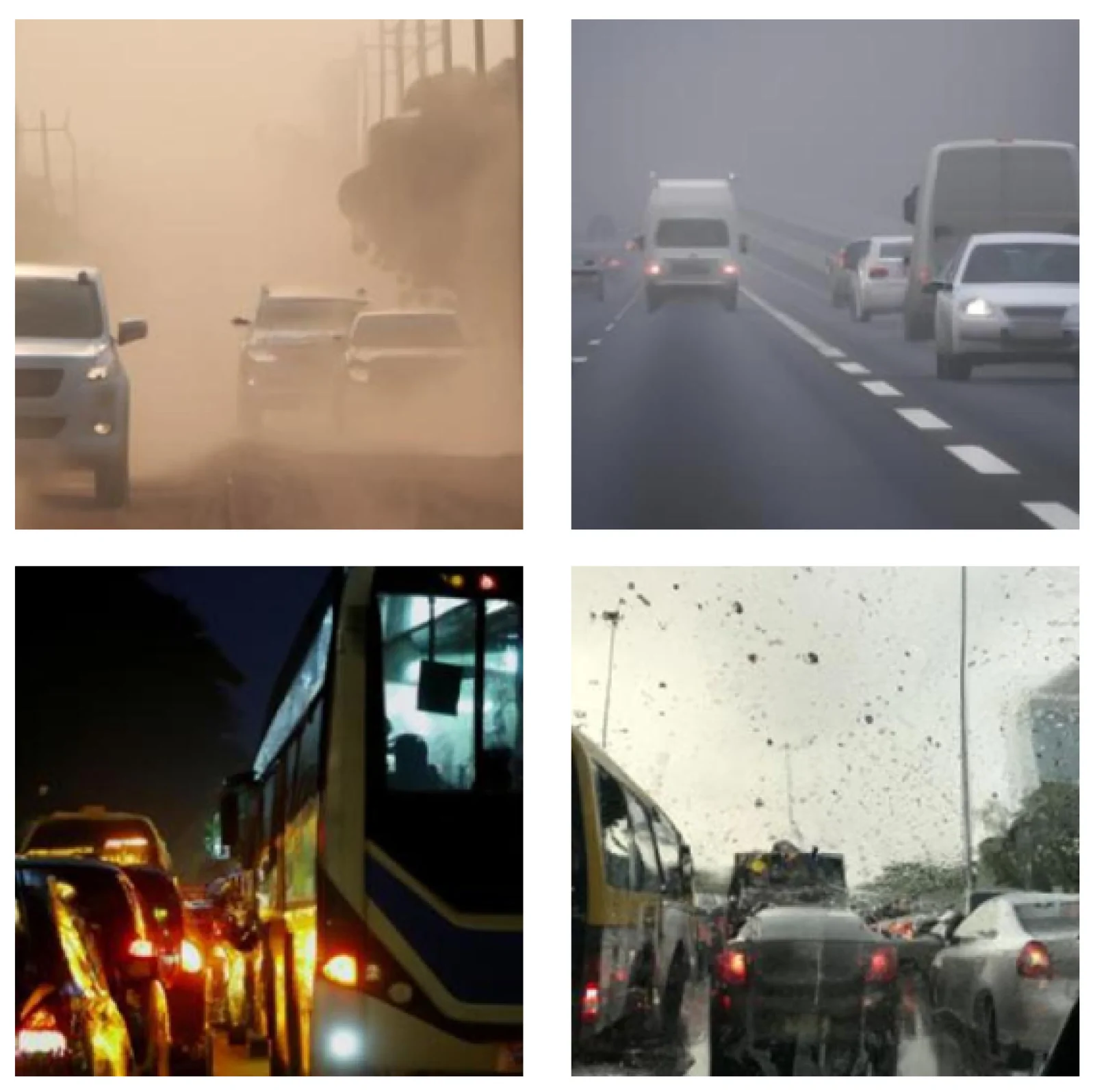
Example images from the WEDGE dataset under various adverse weather conditions.

To further validate the cross-domain generalization capability of the model, this paper selects the WEDGE (WEather images by DALL-E GEneration) dataset [5] for cross-domain generalization experiments. WEDGE is a multi-weather object detection dataset constructed based on a generative vision model, containing a total of 3360 images and 16,513 object annotations, covering 16 weather scenarios, including rain, snow, fog, dust, hurricane, and nighttime, with seven object categories: car, truck, bus, motorcycle, bicycle, person, and van, as shown in Figure 5. Compared with DAWN, WEDGE features a richer variety of weather types and can be used to evaluate the model’s generalization capability under different data distributions and complex weather conditions, thereby validating the applicability of the proposed method in cross-domain scenarios.

Therefore, this paper employs the DAWN dataset [4] to verify the detection performance of the model in real adverse weather scenes, and utilizes the WEDGE dataset [5] to conduct cross-domain generalization experiments, thereby providing a comprehensive evaluation of the proposed method from both detection accuracy and model generalization capability perspectives.

#### 4.1.2. Evaluation Metrics

To comprehensively evaluate the object detection performance of the model under adverse weather conditions, this paper adopts several metrics commonly used in the object detection field, including mAP50-95, mAP50, AP_*s*_, and inference speed (FPS). Specifically, mAP50 denotes the mean average precision across all categories at an Intersection over Union (IoU) threshold of 0.5, mAP50-95 represents the average precision computed over IoU thresholds ranging from 0.50 to 0.95 (with a step size of 0.05), providing a more comprehensive reflection of the model’s detection performance, AP_*s*_ measures the model’s detection capability for small objects, and FPS (Frames Per Second) indicates the number of images the model can process per second, which is used to evaluate its real-time detection capability. The calculation formula for mAP50 is as follows:

(9) $$mAP50=\frac{1}{N}\sum_{i=1}^{N}AP_{i}$$

where *N* denotes the number of object categories, and $AP_{i}$ represents the average precision for the *i*-th category. This paper integrates the above evaluation metrics to conduct a comprehensive analysis of each model’s performance from the perspectives of overall detection accuracy, localization accuracy at different IoU thresholds, small object detection capability, and real-time detection performance.

### 4.2. Main Detection Results and Performance Analysis on the DAWN Dataset

This section presents the detection performance of the proposed WFG-RT-DETR on the DAWN adverse-weather dataset [4] and compares it with several representative object detection methods. The reported results of Faster R-CNN [6], YOLOv11-TWCS [44], AWD-YOLO [37], and YOLOv8m [39] are directly obtained from their corresponding publications and are included as reference values. Since these methods may adopt different training configurations, input resolutions, preprocessing strategies, and implementation environments, their reported results are not regarded as strictly controlled comparisons.

To provide a controlled evaluation of the proposed method, RT-DETR [2] was implemented as the baseline, and WFG-RT-DETR was evaluated under the same experimental settings. The two models use the same dataset split, input resolution, preprocessing procedure, training strategy, and evaluation protocol. Therefore, the performance difference between RT-DETR and WFG-RT-DETR provides the primary quantitative evidence for evaluating the effectiveness of the proposed method.

The experimental results are presented in Table 1.

The quantitative results in Table 1 show the detection performance reported for several representative methods on the DAWN dataset. Since the results of YOLOv11-TWCS [44], AWD-YOLO [37], and YOLOv8m [39] are taken from their corresponding publications, they are mainly used to provide a reference for the relative performance of the proposed method. The controlled comparison is conducted between RT-DETR [2] and WFG-RT-DETR under the same experimental settings.

WFG-RT-DETR achieves a mAP50-95 of 45.03%, compared with 41.69% for the RT-DETR baseline, corresponding to an improvement of 3.34 percentage points. The mAP50 also increases from 64.62% to 68.89%. Furthermore, AP_*s*_, AP_*car*_, and AP_*person*_ improve by 4.72, 4.40, and 5.37 percentage points, respectively. These results indicate that the proposed method improves detection performance under adverse-weather conditions.

On this basis, the proposed WFG-RT-DETR further elevates the mAP50-95 to 45.03%, an increase of 3.34 percentage points over the baseline, while achieving an mAP50 of 68.89%, delivering excellent results across all major metrics. Notably, the small object detection accuracy AP_*s*_ surged from the baseline’s 34.74% to 39.46%. This gain directly benefits from the dual-branch collaborative mechanism of the WFG module: the frequency-domain gating effectively suppresses the obliteration of weak edges by high-frequency noise induced by rain and snow, while the spatial strip convolution compensates for the local contours that might be over-smoothed due to denoising, together achieving high-fidelity extraction of features for small-sized objects. In key categories, the detection accuracy for Car and Person reached 54.60% and 43.50%, respectively, representing improvements of 4.40 and 5.37 percentage points over the baseline, indicating that WFG provides a universal enhancement effect for objects of different scales in traffic scenes.

### 4.3. Computational Complexity Analysis

To quantitatively evaluate the real-time performance of the proposed method, we compare the computational complexity of RT-DETR and WFG-RT-DETR in terms of parameter count, FLOPs, and inference latency. As shown in Table 2, the baseline RT-DETR contains 49.794 M parameters and requires 65.886G FLOPs for a 640×640 input. The proposed WFG-RT-DETR introduces only a marginal increase of 0.205 M parameters and 0.578 G FLOPs. In terms of inference speed, we measure the latency on a single NVIDIA RTX 4090 GPU with batch size 1 and AMP precision. The baseline RT-DETR achieves a latency of 22.655 ms, while WFG-RT-DETR achieves 23.058 ms, corresponding to a small increase of 0.403 ms. These results indicate that the proposed WFG and WAQS modules improve detection accuracy with negligible additional computational cost, maintaining the real-time inference capability required for practical autonomous driving applications.

### 4.4. Cross-Domain Generalization Capability Analysis

To further validate the generalization capability of the proposed model in unknown weather environments, this paper conducts cross-domain experiments in a Zero-shot (zero-shot transfer) manner. Specifically, the model is trained solely on the DAWN dataset [4], and during the testing phase, it is directly evaluated on the WEDGE dataset [5] without any fine-tuning using WEDGE samples. Since the WEDGE dataset encompasses 16 complex weather conditions, including rain, snow, fog, low illumination, and dust, and exhibits a more complex weather distribution and a more pronounced domain shift compared to the DAWN dataset, it can provide a more objective evaluation of the model’s cross-domain detection capability. The experimental results are presented in Table 3. Since DAWN contains six categories whereas WEDGE contains seven categories, with “van” being an additional category in WEDGE, the cross-domain evaluation is conducted exclusively on the six common categories (car, truck, bus, motorcycle, bicycle, and person). This ensures that the evaluation is performed under a consistent label space and avoids the influence of category mismatch.

As can be seen from Table 3, there are significant differences in the detection performance of different object detection models on the WEDGE dataset. The traditional convolutional detector Faster R-CNN [6] achieves only a mAP50 of 45.41% under complex weather conditions, indicating its limited adaptability to image degradation and background interference caused by adverse weather. Single-stage detection models proposed in recent years, such as YOLOv10s [54], YOLOv11s [44], and YOLOv12s [55], although offering high detection speeds, show limited improvement in detection accuracy in cross-domain scenarios, with YOLOv11s achieving a mAP50 of 45.70%, still struggling to effectively handle the domain shift problem caused by complex weather.

In contrast, Transformer-based detection models exhibit better cross-domain adaptation capability. DN-DETR [47] and RT-DETR [2] achieve mAP50 of 50.80% and 50.09%, respectively, indicating that the global modeling mechanism can alleviate the feature degradation problem caused by complex weather to a certain extent. YOLOv8s-WAMNet [38], by introducing a hybrid attention mechanism and multi-scale feature fusion, achieves a mAP50 of 51.90% on the WEDGE dataset, ranking among the better-performing existing methods.

The proposed WFG-RT-DETR achieves a mAP50 of 52.73% on the WEDGE dataset, the highest among all compared methods. This represents an improvement of 2.64 percentage points over the RT-DETR baseline and 0.83 percentage points over YOLOv8s-WAMNet, demonstrating that the proposed method can maintain high detection accuracy in unknown weather environments and possesses strong cross-domain generalization capability.

It is worth noting that in terms of the mAP50-95 metric, YOLOv8s-WAMNet [38] obtains the highest result of 24.96%, while the proposed method achieves 23.44%. It is analyzed that the proposed WFG module primarily optimizes against high-frequency noise and object feature degradation caused by adverse weather, enabling the model to identify objects more accurately and improve object recall, thus, leading to a more significant improvement in the mAP50 metric. However, mAP50-95 adopts multiple IoU thresholds for comprehensive evaluation, demanding higher localization accuracy for object bounding boxes, and thus there remains room for improvement. This result also reveals a limitation of the current model. Although WFG-RT-DETR achieves a higher mAP50 than the compared methods on WEDGE, its mAP50-95 remains lower than that of YOLOv8s-WAMNet. This indicates that the proposed framework is more effective in improving object recognition and recall than in achieving highly precise localization under large domain shifts.

**Figure 6:**
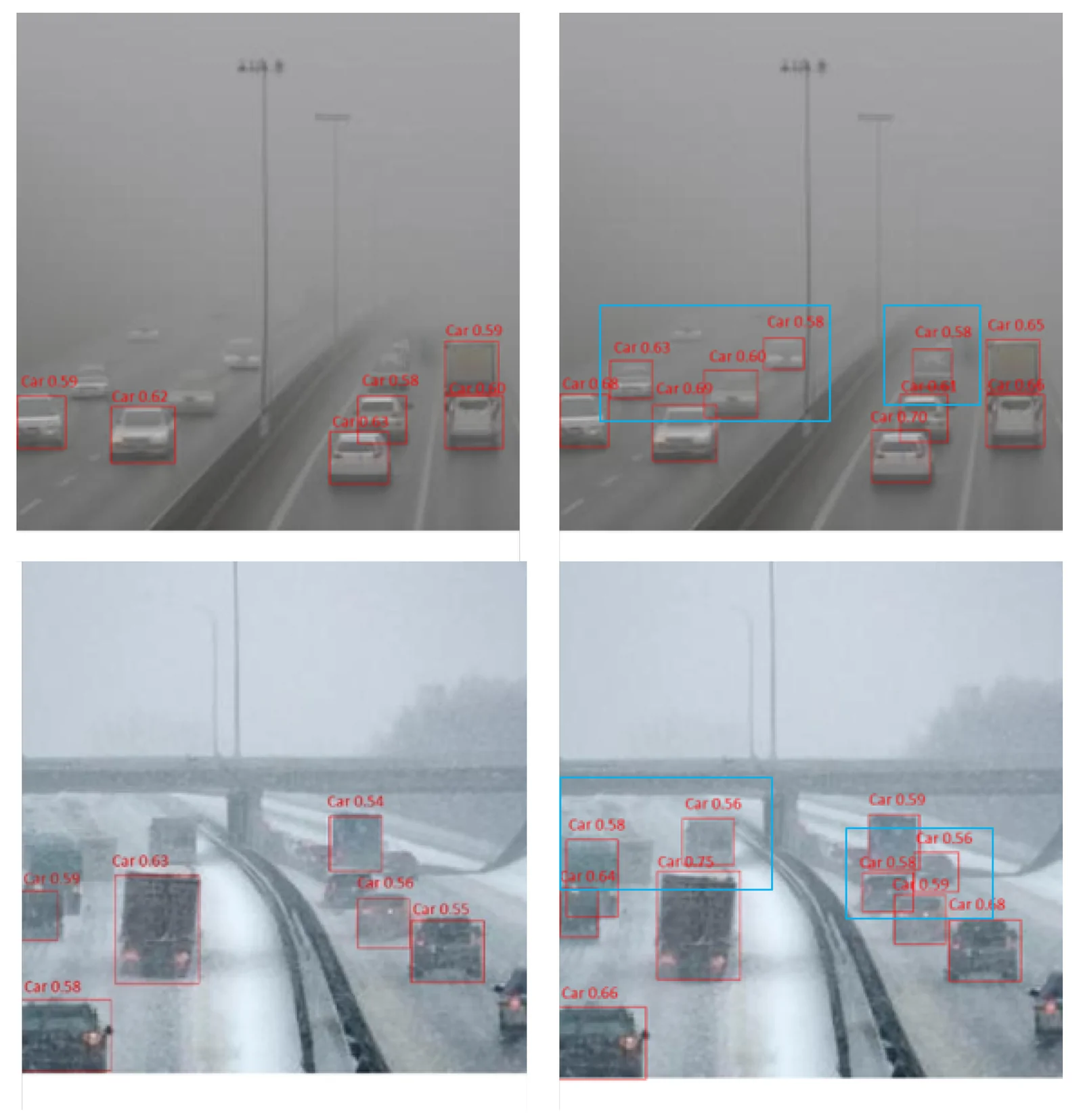
Comparison of detection results between RT-DETR and WFG-RT-DETR on the WEDGE dataset. The blue boxes highlight the missed detections of RT-DETR.

To further intuitively validate the detection capability of the model in unknown weather scenes, this paper selects representative foggy and snowy scenes from the WEDGE dataset and presents a visual comparison of the detection results between RT-DETR and the proposed WFG-RT-DETR, as shown in Figure 6.

As shown in Figure 6, the first column presents the detection results of RT-DETR, while the second column presents the detection results of the proposed WFG-RT-DETR. The first row corresponds to a foggy scene and the second row to a snowy scene. It can be observed that under complex weather conditions, RT-DETR exhibits certain missed detections, particularly in regions with distant objects and low-contrast targets, where some vehicles fail to be correctly detected. In contrast, WFG-RT-DETR is capable of detecting more objects, demonstrating better recognition capability for occluded and distant objects, along with more accurate bounding box localization. This indicates that the proposed method can effectively enhance the detection performance of the model in unknown weather environments.

Taken together, the WFG module effectively strengthens the representation capability of weather-invariant features through frequency-domain adaptive noise suppression and spatial structure compensation, while the WAQS module suppresses false responses caused by environmental factors such as reflections, water puddles, rain, and snow during the query selection stage, thereby reducing false detections in cross-domain scenarios. The two modules work synergistically, enabling the model to achieve a higher mAP50 than current mainstream detection models without any reliance on target domain training data, which demonstrates strong cross-domain generalization potential and robust object detection capability in complex weather conditions, providing a promising foundation for future deployment in real-world adverse weather scenarios.

### 4.5. Ablation Experiments

To verify the effectiveness of each proposed improvement module, ablation experiments are conducted on the DAWN adverse weather dataset [4]. Taking RT-DETR [2] as the baseline model, we evaluate all meaningful combinations of the three modules, including individual contributions and pairwise combinations. The experimental results are presented in Table 4.

As can be seen from Table 4, the baseline RT-DETR model [2] achieves an mAP50-95 of 41.69%, an mAP50 of 64.62%, and a small object detection accuracy AP_*s*_ of 34.74% on the DAWN dataset. After introducing the EMA strategy [3], the model’s mAP50-95 improves to 42.28% and mAP50 to 66.51%, indicating that EMA can effectively mitigate the gradient oscillation problem caused by the distribution fluctuation of adverse weather samples, thereby enhancing the stability of the training process and the model’s generalization capability.

To further evaluate the individual contribution of each module, we also conduct experiments with only WAQS or only WFG added to the baseline. As shown in Table 4, adding WAQS alone improves mAP50-95 from 41.69% to 42.12%, while adding WFG alone achieves a more substantial gain to 43.58%. This indicates that WFG contributes more significantly to the overall performance improvement than WAQS when used independently.

With the further addition of WAQS to the EMA-enhanced baseline (EMA + WAQS), the model’s mAP50-95 increases to 42.65% and AP_*s*_ to 37.50%. Although the overall accuracy gain is relatively modest, the small object detection performance is further improved. This suggests that WAQS can reduce the interference of false targets such as reflective regions and rain and snow noise on the decoder by recalibrating the confidence scores of candidate queries, thereby reducing false positive predictions.

When WFG is further added to the EMA + WAQS configuration, the complete model achieves the best performance of 45.03% mAP50-95, a gain of 2.38 percentage points over the EMA + WAQS configuration; mAP50 rises to 68.89%; and AP_*s*_ improves to 39.46%. The experimental results indicate that WFG effectively filters out high-frequency interference information under adverse weather conditions and enhances the representation capability of object edges and texture features through frequency-domain adaptive noise suppression and spatial structure compensation, thereby significantly improving detection accuracy, especially for small objects and in complex background scenes. The pairwise combination results also show that EMA + WFG (44.12%) and WAQS + WFG (44.46%) consistently outperform EMA + WAQS (42.65%), further confirming the dominant role of WFG in performance improvement.

In summary, the EMA [3], WAQS, and WFG modules all contribute to performance improvement to varying degrees, with WFG making the most significant contribution and serving as the primary source of performance gain in this paper. To further analyze the reasons behind the performance improvement, the next section will provide a qualitative analysis of the proposed method through feature response visualization.

To further investigate the contribution of each sub-component within the WFG module, we conduct additional ablation experiments by independently evaluating the frequency-domain gating branch and the strip-convolution spatial branch. Both variants are initialized from the best-performing WFG-RT-DETR checkpoint and fine-tuned under the same experimental settings. As shown in Table 5, the full WFG model achieves the best overall performance with an AP50 of 0.689 and AP75 of 0.487. The frequency-only branch achieves an AP50 of 0.575 and AP75 of 0.342, outperforming the spatial-only branch, which achieves 0.502 AP50 and 0.356 AP75. These results indicate that the frequency-domain gating mechanism plays a more dominant role in suppressing weather-induced interference, while the spatial branch provides complementary structural enhancement. When both branches are combined, the full WFG module achieves substantial performance gains over either sub-component alone, demonstrating the effectiveness of the dual-branch collaborative design.

### 4.6. Visualization Analysis

#### 4.6.1. Visualization of Detection Results Under Different Weather Conditions

**Figure 7:**
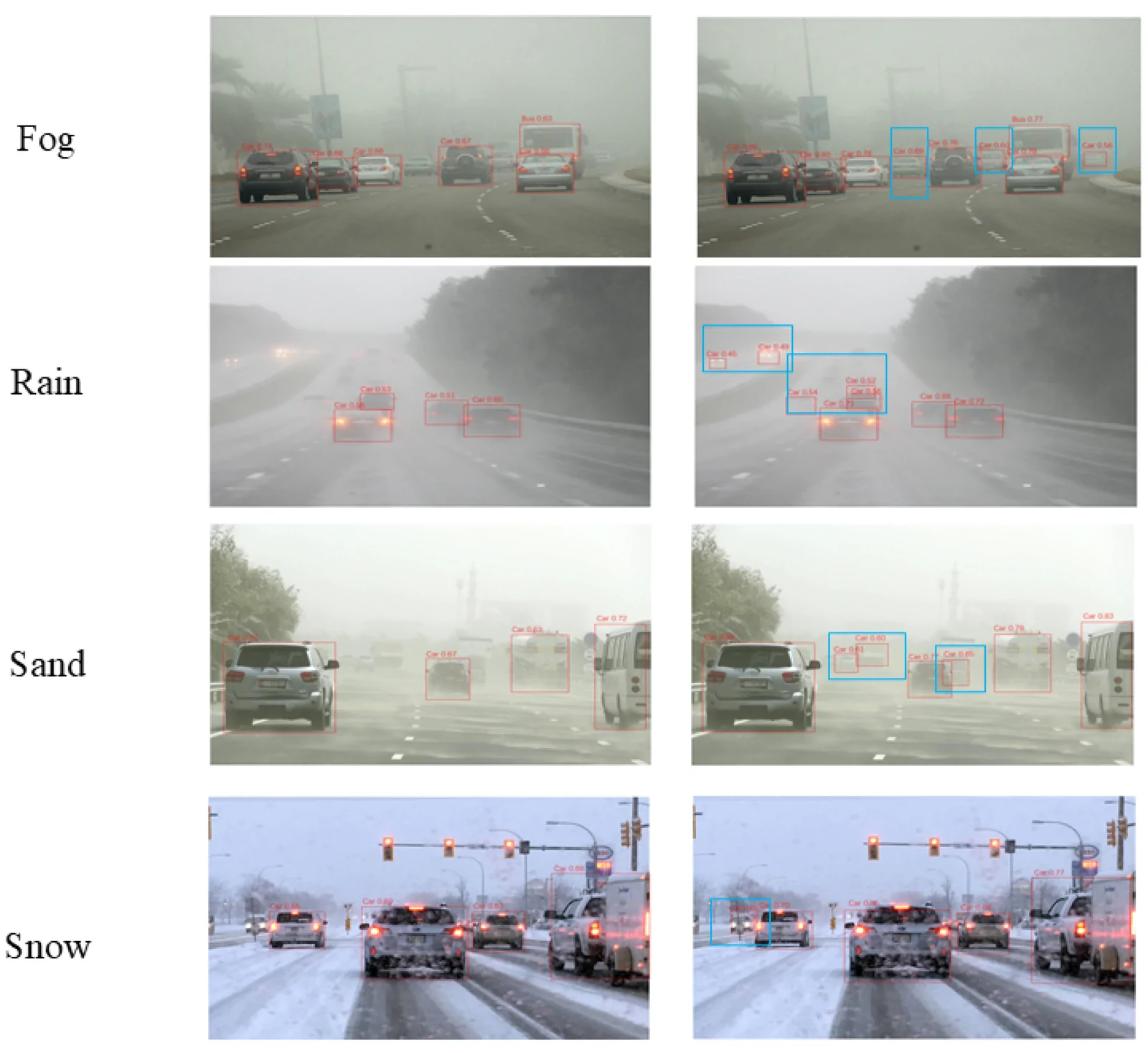
Visual comparison of detection results between RT-DETR and WFG-RT-DETR under different adverse weather conditions on the DAWN dataset. The (**left**) column shows the detection results of RT-DETR, and the (**right**) column shows those of WFG-RT-DETR. The blue boxes highlight the missed detections of RT-DETR.

To further validate the detection performance of the proposed model under different adverse weather scenarios, this paper selects four representative weather conditions from the DAWN dataset, including Fog, Rain, Sand, and Snow. The detection performances of RT-DETR and WFG-RT-DETR under each weather condition are, respectively, computed, and the detection results are visualized for analysis. The detection results of the two models under different weather conditions are presented in Table 6, and the corresponding detection effect comparison is shown in Figure 7.

Table 6 shows that the proposed WFG-RT-DETR achieves superior detection performance over RT-DETR across all four typical adverse weather scenarios, demonstrating the method’s strong weather adaptability and robustness.

Specifically, in the Fog scenario, the AP of WFG-RT-DETR increases from 55.7% to 57.7%, mAP50 from 86.1% to 88.9%, and mAP50-95 from 58.7% to 61.7%. Foggy conditions lead to an overall reduction in image contrast and blurred object edges, while the WFG module effectively suppresses weather noise and restores object structural information, thereby enabling the model to better detect distant objects and low-contrast targets.

In the Rain scenario, WFG-RT-DETR achieves AP, mAP50, and mAP50-95 of 57.0%, 84.1%, and 57.5%, respectively, representing improvements of 1.0, 0.5, and 1.2 percentage points over RT-DETR. This indicates that the proposed method can, to a certain extent, mitigate the interference of rain streaks and road reflections on object features, improving detection stability in complex backgrounds.

In the Sand (dust) scenario, sandstorm conditions cause color shifts, loss of details, and degradation of object textures, significantly increasing detection difficulty. The proposed method achieves the most pronounced improvement in this scenario, with AP increasing from 44.7% to 47.8%, mAP50 from 65.0% to 69.0%, and mAP50-95 from 50.2% to 54.3%. The experimental results demonstrate that the WFG module can effectively enhance object features in degraded images, improving the model’s detection capability under extreme weather conditions.

In the Snow scenario, the elevated background brightness and weakened object textures caused by snow accumulation make RT-DETR prone to missed detections or localization deviations due to background interference. In contrast, WFG-RT-DETR improves AP to 56.2%, mAP50 to 80.0%, and mAP50-95 to 70.3%, indicating that the enhanced model better preserves object structural information and improves detection accuracy for low-contrast targets.

Figure 7 further illustrates the detection results of the two models under the four types of adverse weather scenes. It can be observed that in foggy and sandy/dusty conditions, RT-DETR misses some distant objects and small targets, whereas WFG-RT-DETR detects more valid objects with more complete predicted bounding boxes. In rainy scenes, the improved model effectively reduces interference from rain streaks and background reflections, leading to more accurate bounding box localization. In snowy scenes, WFG-RT-DETR maintains high detection confidence for low-contrast targets, reducing false detections and missed detections caused by background noise, resulting in a more stable overall detection performance.

Taken together, the experimental results from Table 6 and Figure 7 demonstrate that the proposed WFG-RT-DETR outperforms the RT-DETR baseline across all four typical adverse weather conditions. Notably, the improvement in the Sand scenario is the most pronounced, indicating that the proposed WFG module can fully exploit the advantages of frequency-domain noise suppression and spatial structure compensation to effectively enhance the representation of object features under complex weather. Meanwhile, the WAQS module further optimizes the decoder query quality and mitigates the impact of weather noise on the detection process, enabling the model to achieve better detection accuracy and robustness across diverse weather environments. These findings further validate the effectiveness of the proposed method for object detection in adverse weather.

#### 4.6.2. Failure Cases and Limitations

Although WFG-RT-DETR achieves improved detection performance under various adverse weather conditions, it is not completely immune to severe environmental interference. In extremely degraded scenes, some detection failures may still occur. For example, heavy fog and dense snowfall can significantly reduce object contrast and obscure object boundaries, making small or distant objects difficult to distinguish from the background. Strong reflections and irregular weather-related patterns may also introduce abnormal visual responses, which can lead to false detections or missed detections. These cases indicate that the proposed WFG module can suppress part of the weather-induced interference, but cannot completely recover object information that has been severely degraded or lost during image formation.

In addition, the cross-domain results on the WEDGE dataset reveal a limitation in precise object localization. Although WFG-RT-DETR achieves a higher mAP50 than the RT-DETR baseline, its mAP50-95 remains lower than that of some compared methods. Since mAP50-95 evaluates detection performance over multiple IoU thresholds, this result suggests that the proposed method is more effective in improving object recognition and recall than in maintaining highly precise bounding-box localization under large domain shifts. This limitation becomes more apparent for small, distant, or partially occluded objects in complex weather scenes.

Another limitation is that the current framework mainly relies on visible-light images. When visibility is severely degraded by heavy fog, strong precipitation, water splashes, or other extreme weather phenomena, the available visual information may become insufficient for reliable detection. In such situations, frequency-domain feature refinement and weather-aware query selection alone may not be sufficient to fully recover the missing semantic and structural information.

Overall, these failure cases indicate that WFG-RT-DETR still has room for improvement in extreme weather conditions, particularly in highly degraded scenes, strong weather-induced artifacts, and precise localization of small or partially occluded objects. These observations also provide directions for future research, including more robust localization strategies and the integration of complementary modalities to improve detection reliability under severe weather conditions.

#### 4.6.3. Feature Response Visualization Analysis

**Figure 8:**
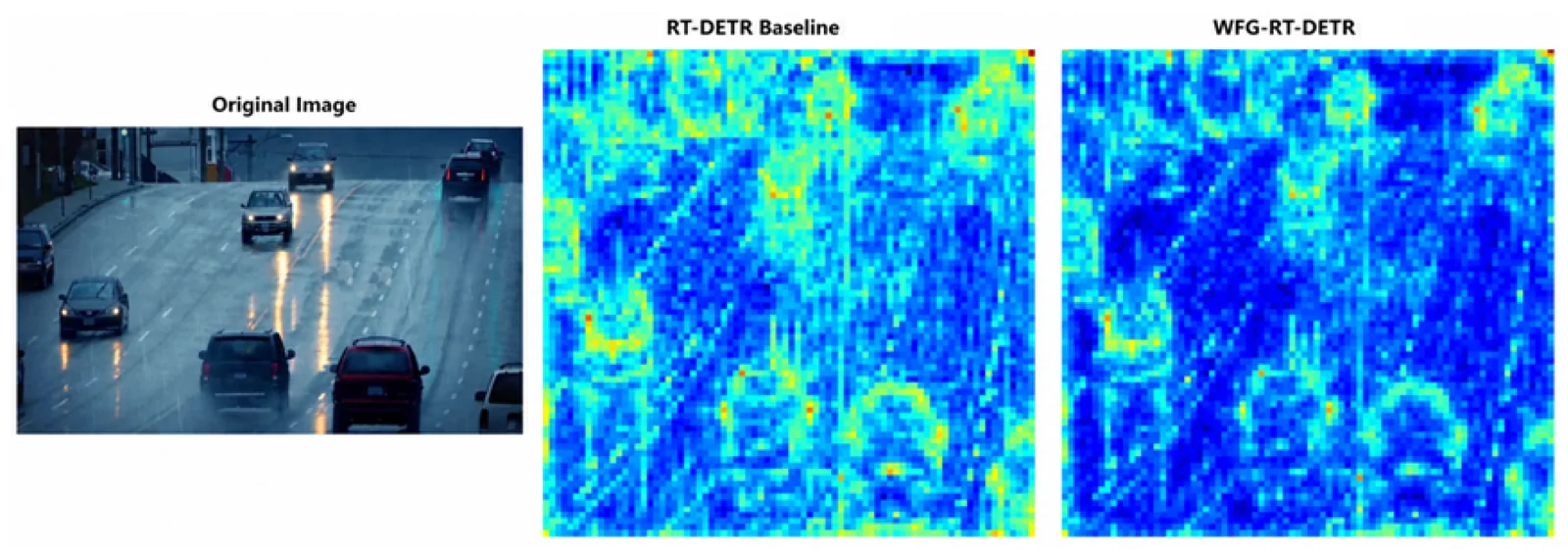
Comparison of feature responses under adverse weather scenes. The color intensity indicates the magnitude of feature response.

To further validate the feature extraction capability of the proposed method under adverse weather conditions, this section selects a typical rainy scene sample from the DAWN dataset and visualizes the intermediate-layer feature responses of the model. Figure 8 presents the original input image, the feature map of the RT-DETR baseline model, and the corresponding feature response results of the proposed WFG-RT-DETR model.

The feature response distribution of the baseline model is relatively scattered, with a large number of randomly activated high-response regions appearing in the feature map. Due to environmental interference such as road reflections, water puddles, and rain streaks in rainy scenes, certain background regions produce strong activations, resulting in an insufficiently pronounced response contrast between object regions and background regions. This indicates that under adverse weather conditions, environmental noise has become involved in the feature representation process, which can easily affect subsequent object localization and classification.

In contrast, the feature maps of WFG-RT-DETR exhibit a more regularized response distribution. It can be observed that the response intensity in large background areas is significantly reduced, with fewer invalid responses in background regions, while object regions maintain strong activations, and random noise activations are effectively suppressed. Meanwhile, the regions where vehicles are located and their surrounding structural information still maintain strong responses, indicating that the model better preserves object-related semantic features while mitigating weather noise interference.

The primary reason for this phenomenon lies in the fact that the WFG module suppresses high-frequency interference such as rain and snow through the frequency-domain adaptive noise gating mechanism and restores object edges and local structural information via the spatial compensation branch, thereby improving the quality of feature representations. Furthermore, the WAQS mechanism further constrains the query point selection in the decoding stage, enabling the network to focus more on genuine object regions rather than environmental noise regions.

Combined with the ablation experiment results in Table 4, it can be found that the reduction of background noise responses in the feature maps exhibits a consistent trend with the improvement in detection accuracy. From a qualitative perspective, these visualization results verify that the proposed WFG-RT-DETR model possesses stronger feature purification and object representation capabilities under adverse weather scenes, providing effective support for the subsequent improvement in detection performance.

## 5. Conclusions

To address the problem that object detection under adverse weather conditions is susceptible to factors such as rain and snow noise, haze occlusion, and illumination variations, which lead to object feature degradation and reduced detection performance, this paper proposes WFG-RT-DETR, an object detection model tailored for complex weather scenes built upon RT-DETR. While preserving the advantages of the end-to-end detection framework, the proposed method introduces a Weather-Focused Guidance (WFG) module to perform frequency-domain denoising and spatial structure enhancement on features, designs a Weather-Aware Query Selection (WAQS) mechanism to optimize candidate queries, and incorporates an EMA strategy to improve model training stability. Experimental results demonstrate that the proposed method outperforms the RT-DETR baseline on the DAWN dataset, achieving significant improvements in metrics, such as mAP50, mAP50-95, and small object detection accuracy. Meanwhile, cross-domain testing results on the WEDGE dataset further validate that the model possesses strong generalization capability and adaptability to complex weather conditions. Nevertheless, several limitations remain. The current framework mainly relies on visible-light images and may still suffer from severe information degradation under extreme weather conditions, such as heavy fog, strong precipitation, water splashes, and hail. In addition, although WFG improves the robustness of feature representation, precise localization under large domain shifts remains challenging, as reflected by the relatively lower mAP50-95 performance on the WEDGE dataset. Future work will further investigate more robust frequency-domain modeling and weather-aware query mechanisms for extreme weather scenarios. In particular, weather-induced artifacts such as water splashes on flooded roads and hail can introduce dense and irregular visual changes, which may lead to false detections in traffic monitoring systems. Wiseman [56] reported similar weather-induced interference in real-time traffic congestion monitoring. These observations motivate extending the proposed frequency-domain gating and weather-aware query selection mechanisms toward more robust camera-based traffic monitoring under extreme adverse conditions. In addition, the fusion of complementary modalities, such as infrared images and LiDAR, with visible images will be explored to improve detection reliability under severe weather and low-illumination conditions.

## Figures and Tables

**Table 1:** Comprehensive comparison of performance metrics of various models on the DAWN dataset.

Model	mAP50-95	mAP50	AP_*s*_	AP_*car*_	AP_*person*_
YOLOv11-TWCS [44]	36.10%	59.10%	–	–	–
AWD-YOLO [37]	40.80%	67.70%	32.10%	–	–
YOLOv8m [39]	–	58.00%	–	–	–
RT-DETR (baseline)	41.69%	64.62%	34.74%	50.20%	38.13%
**WFG-RT-DETR (Ours)**	**45.03%**	**68.89%**	**39.46%**	**54.60%**	**43.50%**

**Table 2:** Computational complexity comparison between RT-DETR and WFG-RT-DETR.

Model	Params (M)	FLOPs (G)	Latency (ms)	FPS
RT-DETR (baseline)	49.794	65.886	22.655	44.1
WFG-RT-DETR (Ours)	49.999	66.464	23.058	43.4

**Table 3:** Comparison of detection results across different models on the WEDGE dataset.

Model	mAP50	mAP50-95
Faster R-CNN (ResNet50) [6]	45.41%	–
YOLOv10s [54]	38.80%	18.70%
YOLOv11s [44]	45.70%	23.20%
YOLOv12s [55]	44.60%	22.12%
DN-DETR [47]	50.80%	24.30%
YOLOv8s-WAMNet [38]	51.90%	24.96%
RT-DETR [2]	50.09%	22.20%
**Ours (WFG-RT-DETR)**	**52.73%**	**23.44%**

**Table 4:** Results of ablation experiments on the DAWN dataset.

Baseline	EMA	WAQS	WFG	mAP50-95 (%)	mAP50 (%)	AP_*s*_ (%)
✓				41.69	64.62	34.74
✓	✓			42.28	66.51	37.10
✓		✓		42.12	65.34	36.20
✓			✓	43.58	67.45	38.20
✓	✓	✓		42.65	65.39	37.50
✓	✓		✓	44.12	67.86	38.72
✓		✓	✓	44.46	68.22	39.05
✓	✓	✓	✓	45.03	68.89	39.46

Checkmarks (✓) denote that the corresponding module is adopted in the model.

**Table 5:** Ablation study on WFG sub-components on the DAWN dataset.

Model	mAP50 (%)	mAP50-95 (%)
WFG (Full)	0.689	0.487
Freq-only	0.575	0.342
Spatial-only	0.502	0.356

**Table 6:** Comparison of detection results between RT-DETR and WFG-RT-DETR under different weather conditions in DAWN.

Model	Weather	AP	mAP50	mAP50-95
RT-DETR	Fog	55.7%	86.1%	58.7%
	Rain	56.0%	83.6%	56.3%
	Sand	44.7%	65.0%	50.2%
	Snow	54.6%	77.8%	67.9%
WFG-RT-DETR	Fog	**57.7%**	**88.9%**	**61.7%**
	Rain	**57.0%**	**84.1%**	**57.5%**
	Sand	**47.8%**	**69.0%**	**54.3%**
	Snow	**56.2%**	**80.0%**	**70.3%**

## Data Availability

The DAWN dataset is publicly available at https://data.mendeley.com/datasets/766ygrbt8y/3 (accessed on 15 May 2026) (https://doi.org/10.17632/766ygrbt8y.3). The WEDGE dataset is publicly available at https://github.com/Infernolia/WEDGE (accessed on 15 May 2026) under the CC BY-NC-SA 4.0 license.
